# Characterization of Cytokines and Proliferation Marker Ki-67 in Chronic Rhinosinusitis with Recurring Nasal Polyps

**DOI:** 10.3390/arm90050053

**Published:** 2022-10-14

**Authors:** Rudolfs Janis Viksne, Gunta Sumeraga, Mara Pilmane

**Affiliations:** 1 Department of Otorhinolaryngology, Riga Stradins University, Pilsonu Street 13, LV-1002 Riga, Latvia; 2 Daugavpils Regional Hospital, Vasarnicu Street 20, LV-5417 Daugavpils, Latvia; 3 Pauls Stradins Clinical University Hospital, Pilsonu Street 13, LV-1002 Riga, Latvia; 4 Institute of Anatomy and Anthropology, Riga Stradins University, Kronvalda Boulevard 9, LV-1010 Riga, Latvia

**Keywords:** rhinosinusitis, nasal polyps, proliferation marker, cytokines

## Abstract

**Highlights:**

**What are the main findings?**

- Cytokines are found in greater numbers in subepithelial connective tissue rather than epithelial cells in nasal polyps.
- IL-6 is an important factor in the pathogenesis of recurrent nasal polyps.

**What are the implications of the main finding?**

- IL-6 was established as an important factor for further research in CRSwNP
- Possible future clinical implications, yet further research is necessary.

**Abstract:**

Background: Chronic rhinosinusitis with nasal polyps (CRSwNP) is a chronic inflammation of the mucosa of the nose and paranasal sinuses with the presence of polyps, affecting between 2.7% and 4.4% of the population. Cytokine analysis has become important in research on inflammatory mechanisms in CRSwNP. Therefore, our aim is to investigate the complex appearance, relative distribution, and interlinks of IL-1, IL-4, IL-6, IL-7, IL-8, IL-10, IL-12, and Ki-67 in CRSwNP. Methods: Samples of nasal polyps were obtained from 19 patients with previously diagnosed CRSwNP and the recurrence of polyps after previous surgeries. The control group consisted of samples from 17 otherwise healthy individuals with isolated nasal septum deviations. Tissues were stained for previously mentioned cytokines and Ki-67 immunohistochemically. Results: Polyp samples showed an increased presence of cytokines in subepithelial connective tissue and a decreased appearance in epithelium when compared to controls. There were several very strong, strong, and moderate correlations among factors. Conclusions: IL-6 strongly correlates with other cytokines as well as with the proliferation marker Ki-67, which suggests significant stimulation of this regulatory cytokine and its possible involvement in the pathogenesis of recurrent nasal polyps. IL-4, IL-7, IL-10, and IL-12 correlate with Ki-67, which suggests the possible involvement of these cytokines in tissue cell proliferation in the case of recurrent nasal polyps.

## 1. Introduction

Chronic rhinosinusitis (CRS) is an inflammation of the nasal cavity and paranasal sinuses that causes unpleasant symptoms, such as nasal congestion or blockage, nasal discharge, facial pain, pressure, and reduction or loss of smell, for more than twelve weeks and significantly decreases quality of life as well as impairs productivity [1,2]. CRS is estimated to have a prevalence ranging from 4.5% to 12% in North American and European countries [1]. This disease is further divided into chronic rhinosinusitis with nasal polyps (CRSwNP) and chronic rhinosinusitis without nasal polyps (CRSsNP). The presence of nasal polyps in chronic rhinosinusitis is associated with even more significant morbidity and a decreased quality of life [3]. It is estimated that CRSwNP affects between 2.7% and 4.4% of the population [2]. Management of CRS with nasal polyps revolves around the long-term use of intranasal corticosteroids, saline rinses, and additional possibilities such as oral corticosteroids, macrolide antibiotics, and biologic medication [2]. Surgical nasal polyp management provides an important treatment option after exhausting conservative possibilities, yet is not curative, as approximately 40% of patients experience recurrence of polyposis 6 months after surgery. The high rate of recurrence prompts further research into the pathogenesis and management of CRSwNP, as there have not been many significant breakthroughs in the surgical and medical treatment of this disease in the last decade [4]. 

Initially, viral and bacterial causative agents were thought to be behind the aetiology of chronic rhinosinusitis [5]. As it does not exclude the possibility of infectious CRSwNP, for a while now, identification of the inflammatory mechanisms taking place in the tissue of CRS has diverted the attention from determining the alethiological causes. Ongoing investigations are leading with a hypothesis that these various endotypes are a result of dysfunctional interactions between the host tissue and environmental stressors at the mucosal level [2]. 

**Figure 1:**
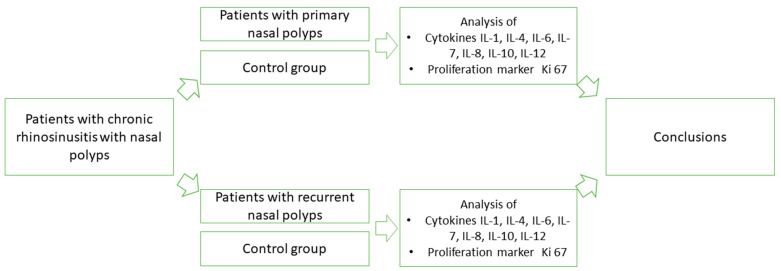
Schematic overview of analysis of primary and recurrent nasal polyps. *Abbreviations:* Ki-67—proliferation marker; IL-1—interleukin 1 alpha; IL-4—interleukin 4; IL-6—interleukin 6; IL-7—interleukin 7; IL-8—interleukin 8; IL-10—interleukin 10; IL-12—interleukin 12.

Tissue cytokines are currently the most used biomarkers in the analysis of the pathogenetic mechanisms of chronic rhinosinusitis [6]. Interleukin 1 alpha (IL-1α) is a member of Interleukin 1 family. It functions as a pro-inflammatory cytokine. IL-1α precursor is present in all cell types and is released upon cell necrosis as a bioactive mediator and an alarmin [7]. Interleukin 4 (IL-4) is one of the central cytokines in allergic inflammation. This type of inflammation is characterized by eosinophilia, elevated IgE levels, and increased mucous production. It is a signature cytokine of type 2 inflammation that is initiated by an allergen or a parasite [8]. Interleukin 6 (IL-6) is a family of cytokines that consists of IL-6 and various other members (for example interleukin 11, interleukin leukemia inhibitory factor, and interleukin 27). Moreover, it characterizes a non-type 2 inflammation which is also associated with CRS. Interleukin 7 (IL-7) has profound growth-promoting effects on progenitors of B cells. It has a role in regulating the development of various immune cells such as T lymphocytes, B lymphocytes, and natural killer cells [9]. Interleukin 8 (IL-8) is a prototypical chemokine that directs cell migration to the site of inflammation. Secretion of IL-8 is principally ensured by monocytes and macrophages, although it is made by various cells [10]. Interleukin 10 (IL-10) is an anti-inflammatory cytokine, which is secreted to downregulate the excessive inflammatory effects of other cytokines [11]. Interleukin 12 (IL-12) is a heterodimeric pro-inflammatory cytokine produced by dendritic cells, macrophages, and B cells in response microbial pathogens. It helps in forming a link between adaptive and innate immunity [12]. In addition to cytokines, the proliferation marker Ki-67 is worth examining. Ki-67 correlates with cytokines, which could be helpful in determining which of the interleukins participates in the facilitation of tissue proliferation. It has not been evaluated extensively in the context of CRS, yet we discussed its possible indication of cell proliferation in our previous work [13]. As previously mentioned, a considerable number of patients experience recurrence of nasal polyps after a while, requiring revision surgery [14]. In our previous study, we have evaluated the mentioned cytokines and the proliferation marker Ki-67 in the tissue of primary nasal polyps without samples of recurring CRSwNP. We found significant differences between a decreased factor presence in the epithelium and an increased presence in the subepithelial connective tissue of nasal polyps compared to the control group [13] (Figure 1). 

Considering role of cytokines in the research on chronic rhinosinusitis and the Ki-67 appearance during cell proliferation and significant rates of nasal polyp recurrence, the aim of this work was to investigate the complex appearance, relative distribution, and interlinks of IL-1, IL-4, IL-6, IL-7, IL-8, IL-10, IL-12, and Ki-67 in CRSwNP-affected human nasal mucosa in the case of recurrent nasal polyps after previous surgery.

## 2. Materials and Methods

### 2.1. Patients

The study group consisted of 19 individuals that were diagnosed with chronic rhinosinusitis with nasal polyps (CRSwNP) and had previously undergone functional endoscopic sinus surgery (FESS) yet experienced a recurrence of nasal polyps after a while. Constituting the group were 10 female and 9 male patients from whom nasal polyp samples were taken during FES surgery. Patients’ ages ranged from 31 to 88 years with average of 55.4 (SD ± 17) years. Patients with coagulopathies, immunodeficiencies, or exacerbation of CRS symptoms 2 weeks prior to surgery were excluded from the study. The control group consisted of inferior turbinate mucosa samples from 17 otherwise healthy individuals whose ages ranged from 25 to 51 years with an average age of 36.5 (SD ± 7.9) years, who were diagnosed with isolated nasal septum deviations and no prior diagnosis of CRS. Individuals with coagulopathies and immunodeficiencies were excluded. Research was reviewed and approved by the ethics committee of Riga Stradins University (6-1/10/59. 26 October 2020). Information about the study was presented, and patients voluntarily agreed to take part. A written consent was obtained. 

### 2.2. Morphological Methods

Samples were initially collected in a mixture of 2% formaldehyde and 0.2% picric acid in 0.1 M phosphate buffer (pH 7.2) for up to 72 h. After that, they were rinsed in Tyrode’s buffer (content: NaCl, KCl, CaCl_2_·2H_2_O, MgCl_2_·6H_2_O, NaHCO3, NaH_2_PO_4_·H_2_O, and glucose) containing 10% saccharose for 12 h and then embedded into paraffin. Three-micrometer thin sections were cut and then stained with hematoxylin and eosin for morphological routine evaluation. The biotin–streptavidin biochemical method was used for immunohistochemistry (IMH) to detect: Ki-67 (1325506A, 1:100, Cell Marque, Rocklin, CA, USA), IL-1 α (orb308737, 1:100, Biorbyt, Cambridge, UK), IL-4 (orb10908, 1:100, Biorbyt, Cambridge, UK), IL-6 (sc-130326, 1:100, Santa Cruz Biotechnology Inc., Dallas, TX, USA), IL-7 (orb13506, 1:100, Biorbyt, Cambridge, UK), IL-8 (orb39299, 1:100, Biorbyt), IL-10 (250713, 1:100, BioSite, Täby, Sweden), and IL-12 (orb10894, 1:100, Biorbyt). The slides were analyzed using light microscopy. The results were evaluated with the semi-quantitative counting method to detect positively stained cells in the visual field. Structures in the visual field were categorized as follows: no positive structures in the visual field were labelled as 0, occasional positive structures were labelled with 0/+, few positive structures: +, few-to-moderate number of positive structures in the visual field: +/++, moderate number of positive structures in the visual field: ++, moderate-to-numerous positive structures in the visual field: ++/+++, numerous positive cells in the visual field: +++, numerous-to-abundant structures in the visual field: +++/++++, and abundant positive structures in the visual field was labelled as ++++ [13].

### 2.3. Statistics

Data analysis was conducted using SPSS software (Version 26.0 IBM Corp. Chicago, IL, USA). The results from the semi-quantitative evaluation were transformed into numerical form to perform statistical analysis: 0 to 0; 0/+ to 0.5; + to 1; +/++ to 1.5; ++ to 2; ++/+++ to 2.5; +++ to 3, and ++++ to 4. Spearman’s rank correlation coefficient was used for the nonparametric measuring of the rank correlation—the statistical dependence of ranking between two factors in CRSwNP-affected mucosa. Coefficients with a value ≥0.81 meant a very strong correlation between two variables, 0.61–0.80 meant a strong correlation, 0.41–0.60 meant a moderate correlation, 0.21–0.40 meant a weak correlation, and 0.01–0.20 meant no correlation. The Mann–Whitney U test was used as a nonparametric test to compare significant differences between the recurrent nasal polyp samples and the control group.

## 3. Results

**Figure 2:**
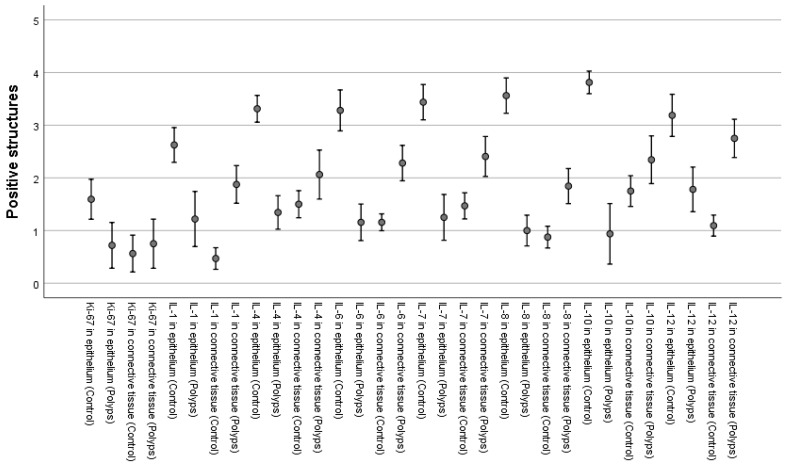
Cytokines and proliferation marker Ki-67 positive structures in samples of recurrent nasal polyps. *Abbreviations:* Ki-67—proliferation marker; IL-1—interleukin 1 alpha; IL-4—interleukin 4; IL-6—interleukin 6; IL-7—interleukin 7; IL-8—interleukin 8; IL-10—interleukin 10; IL-12—interleukin 12; 0/+—occasional positive structures, +—few positive structures, +/++—few-to-moderate number of positive structures, ++—moderate number of positive structures, ++/+++—moderate-to-numerous positive structures, +++—numerous positive cells, +++/++++—numerous-to-abundant structures, ++++—abundance of positive structures in the visual field.

Immunohistochemistry data showed a contrast between the number of positive structures in epithelial cells and subepithelial connective tissue in both the control and nasal polyp samples. Statistically significant differences were observed between the number of positive structures in epithelial cells and subepithelial connective tissue. IL-1α, IL-4, IL-6, IL-8, IL-10, IL-12 (*p* < 0.001), and Ki-67 (*p* = 0.001) were decreased in the epithelium of the nasal polyp samples in comparison to the control samples. IL-1α, IL-6, IL-8, IL-10, IL-12 (*p* < 0.001), and IL-4 (*p* = 0.038) were increased in the subepithelial connective tissue of the nasal polyp samples when compared to the control samples. Ki-67 demonstrated no significant difference (*p* = 0.400) in the connective tissue between the control and the nasal polyp samples. The results from the semi-quantitative evaluation were transformed into numerical form to perform statistical analysis: 0 to 0; 0/+ to 0.5; + to 1; +/++ to 1.5; ++ to 2; ++/+++ to 2.5; +++ to 3, and ++++ to 4 (Figure 2). 

### 3.1. Interleukin 1 Alpha

**Figure 3:**
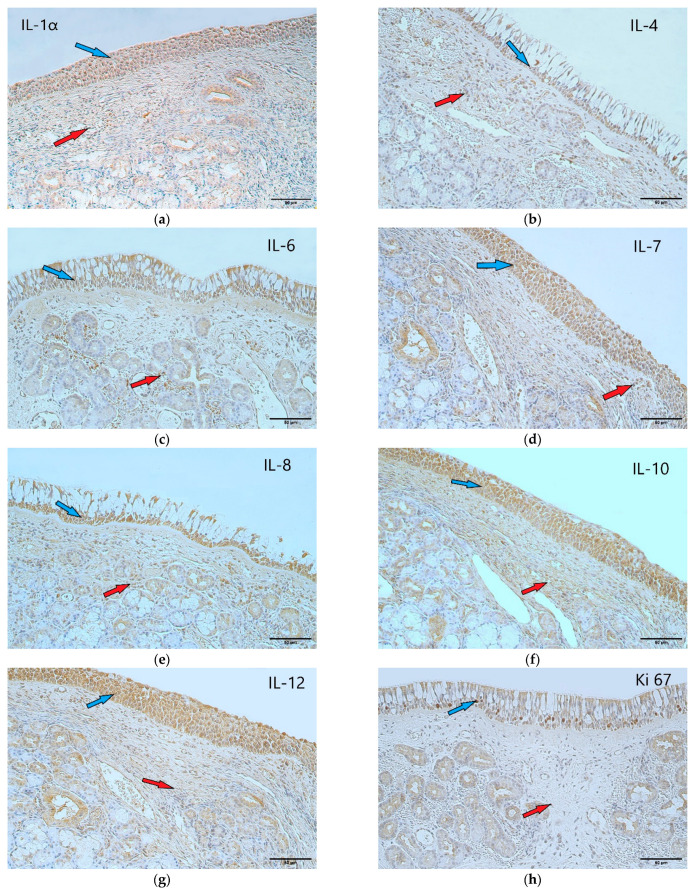
(**a**–**h**) Immunohistochemical micrographs of the control subjects. (**a**) Note moderate-to-numerous IL-1α positive structures in the epithelium (blue arrow) as well as moderate positive structures in the connective tissue (red arrow) of the control sample of normal nasal mucosa. IL-1α IMH, X 200; (**b**) moderate-to-numerous IL-4 positive structures in the epithelium (blue arrow) and moderate positive structures in the subepithelial connective tissue (red arrow) of a control sample. IL-4 IMH, X 200; (**c**) numerous IL-6 positive structures in the control sample epithelium (blue arrow) and moderate positive structures in the connective tissue (red arrow). IL-6 IMH, X 200; (**d**) numerous IL-7 positive structures in the epithelium (blue arrow) of a control sample with few-to-moderate positive structures in the connective tissue (red arrow); almost all epithelial cells are factor positive. IL-7 IMH, X 200; (**e**) note numerous-to-abundant IL-8 positive structures in the epithelium (blue arrow) of the control sample and moderate positive structures in the connective tissue (red arrow). IL-8 IMH, X 200; (**f**) note numerous positive structures in the epithelium (blue arrow) and a moderate number of IL-10 positive structures in the connective tissue (red arrow) of the control sample. IL-10 IMH, X 200; (**g**) numerous IL-12 positive structures in the epithelium (blue arrow) of the control sample, but few-to-moderate positive structures in the connective tissue (red arrow). IL-12 IMH, X 200; (**h**) few-to-moderate number of Ki-67 positive cells in the epithelium (blue arrow) and no positive structures in the connective tissue (red arrow) of the control sample. Ki-67 IMH, X 200.

**Figure 4:**
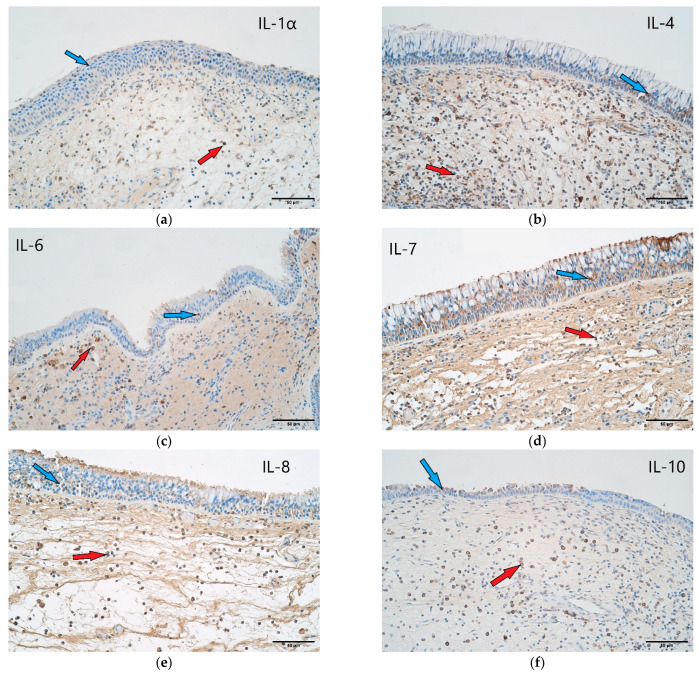

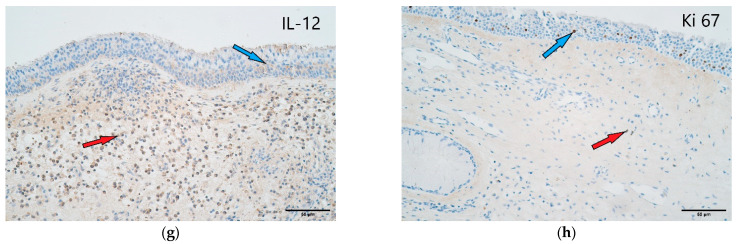
(**a**–**h**) Immunohistochemical micrographs of patients with recurrent nasal polyps. (**a**) Recurrent nasal polyp sample with few IL-1α positive structures in the epithelium (blue arrow) and a moderate number of positive structures in the connective tissue (red arrow). IL-1α IMH, X 200; (**b**) a sample of a recurrent nasal polyp showing moderate IL-4 positive structures in the epithelium (blue arrow) and moderate IL-4 positive structures in the connective tissue (red arrow). IL-4 IMH, X 200; (**c**) few IL-6 positive structures in the epithelium (blue arrow) and moderate IL-6 positive structures in the connective tissue (red arrow) of a nasal polyp. IL-6 IMH, X 200; (**d**) few positive structures in the epithelium (blue arrow) and moderate IL-7 positive structures in the connective tissue (red arrow) of a nasal polyp. IL-7 IMH, X 200; (**e**) nasal polyp sample has few IL-8 positive structures in the epithelium (blue arrow) but a moderate number of positive structures in the connective tissue (red arrow). IL-8 IMH, X 200; (**f**) a sample of a nasal polyp with few IL-10 positive structures in the epithelium (blue arrow) and moderate-to-numerous numbers of IL-10 positive cells in the connective tissue (red arrow). IL-10 IMH, X 200; (**g**) few IL-12 positive structures in the epithelium (blue arrow) and numerous positive cells in the connective tissue (red arrow) of a nasal polyp. IL-12 IMH, X 200; (**h**) occasional Ki-67 positive structures in the epithelium (blue arrow) and few positive cells in the connective tissue (red arrow) of a nasal polyp. Ki-67 IMH, X 200.

When evaluating the IL-1α appearance, it was noted that the control group samples mainly displayed moderate-to-numerous (++/+++) positive structures in the epithelium and occasional (0/+) positive structures in the connective tissue (Figure 2 and Figure 3a). In contrast, the nasal polyp samples displayed only a few (+) positive structures in the epithelial cells but moderate (++) numbers in the subepithelial connective tissue (Figure 2 and Figure 4a).

Epithelial IL-1α had a very strong positive correlation with epithelial IL-6 (R = 0.821, *p* < 0.001) (Table 1), a strong positive correlation with epithelial IL-12 (R = 0.796, *p* < 0.001), connective tissue IL-12 (R = 0.740, *p* < 0.001), epithelial IL-10 (R = 0.699, *p* = 0.001), epithelial IL-7 (R = 0.690, *p* = 0.002), epithelial IL-4 (R = 0.688, *p* = 0.001), connective tissue IL-4 (R = 0.675, *p* = 0.002), epithelial Ki-67 (R = 0.647, *p* = 0.003), and connective tissue Ki-67 (R = 0.625, *p* = 0.004) (Table 2) and a moderate correlation with connective tissue IL-1α (R = 0.580, *p* = 0.009) and epithelial IL-8 (R = 0.526, *p* = 0.021) (Table 3).

Connective tissue IL-1α had a strong positive correlation with connective tissue IL-12 (R = 0.696, *p* = 0.001) (Table 2).

### 3.2. Interleukin 4

In the case of IL-4, numerous (+++) positive structures were seen in the epithelium and few-to-moderate (+/++) in the connective tissue of the control samples (Figure 2 and Figure 3b). Nasal polyp samples again displayed a contrast with few-to-moderate (+/++) positive structures in the epithelial layer and moderate (++) positive structures in the subepithelial connective tissue (Figure 2 and Figure 4b).

Epithelial IL-4 had a strong positive correlation with epithelial IL-6 (R = 0.781, *p* < 0.001), epithelial IL-12 (R = 0.706, *p* = 0.001), epithelial IL-7 (R = 0.698, *p* = 0.001), connective tissue IL-4 (R = 0.647, *p* = 0.003), epithelial IL-10 (R = 0.636, *p* = 0.003), connective tissue Ki-67 (R = 0.629, *p* = 0.004), and epithelial Ki-67 (R = 0.653, *p* = 0.002) (Table 2) and a moderate positive correlation with connective tissue IL-6 (R = 0.550, *p* = 0.015) (Table 3).

Connective tissue IL-4 had a strong positive correlation with connective tissue IL-7 (R = 0.654, *p* = 0.002), connective tissue IL-6 (R = 0.658, *p* = 0.002), and epithelial IL-1 α (R = 0.657, *p* = 0.002) (Table 2) and moderate correlation with epithelial IL-10 (R = 0.552, *p* = 0.014), epithelial IL-12 (R = 0.545, *p* = 0.016), connective tissue Ki-67 (R = 0.532, *p* = 0.019) epithelial IL-7 (R = 0.530, *p* = 0.024), and epithelial IL-6 (R = 0.468, *p* = 0.043 (Table 3). 

### 3.3. Interleukin 6

IL-6 positive structures were observed in numerous (+++) numbers in the control sample epithelium (Figure 2 and Figure 3c) and in few (+) numbers in the nasal polyp epithelium (Figure 2 and Figure 4c). The control sample connective tissue showed few (+) positive structures (Figure 2 and Figure 3c), whereas the nasal polyp subepithelial connective tissue displayed moderate (++) positive structures (Figure 2 and Figure 4c).

Epithelial IL-6 had a very strong positive correlation with epithelial IL-7 (R = 0.830, *p* < 0.001), epithelial IL-12 (R = 0.826, *p* < 0.001), and epithelial IL-1α (R = 0.821, *p* < 0.001) (Table 1). Epithelial IL-6 also had a strong positive correlation with epithelial IL-4 (R = 0.781, *p* < 0.001) and epithelial Ki-67 (R = 0.647, *p* = 0.003) (Table 2) and a moderate positive correlation with connective tissue IL-12 (R = 0.559, *p* = 0.013), connective tissue Ki-67 (R = 0.559, *p* = 0.013), epithelial IL-10 (R = 0.539, *p* = 0.017), epithelial IL-8 (R = 0.528, *p* = 0.020), connective tissue IL-6 (R = 0.485, *p* = 0.035), and connective tissue IL-4 (R = 0.468, *p* = 0.043) (Table 3). 

Connective tissue IL-6 had a strong positive correlation with epithelial IL-7 (R = 0.663, *p* = 0.003), connective tissue IL-7 (R = 0.661, *p* = 0.002), connective tissue IL-4 (R = 0.658, *p* = 0.002), and epithelial IL-12 (R = 0.615, *p* = 0.005) (Table 2) and a moderate correlation with epithelial IL-4 (R = 0.550, *p* = 0.015), epithelial IL-1α (R = 0.522, *p* = 0.022), and connective tissue IL-12 (R = 0.494, *p* = 0.032) (Table 3).

### 3.4. Interleukin 7

In the case of IL-7, control samples showed numerous (+++) positive structures in the epithelium and few-to-moderate (+/++) positive structures in the connective tissue (Figure 2 and Figure 3d). The nasal polyp samples displayed a few (+) positive structures in the epithelial cells and moderate-to-numerous (++/+++) structures in the connective tissue (Figure 2 and Figure 4d).

Epithelial IL-7 had a very strong positive correlation with epithelial IL-6 (R = 0.830, *p* < 0.001) (Table 1), a strong positive correlation with epithelial IL-12 (R = 0.771, *p* < 0.001), epithelial IL-4 (R = 0.698, *p* = 0.001), epithelial IL-1α (R = 0.690, *p* = 0.002), connective tissue Ki-67 (R = 0.481, *p* = 0.043), and connective tissue IL-6 7 (R = 0.663, *p* = 0.003) (Table 2) and a moderate positive correlation with connective tissue IL-12 (R = 0.547, *p* = 0.019), connective tissue IL-4 (R = 0.530, *p* = 0.024), epithelial Ki-67 (R = 0.481, *p* = 0.043), and connective tissue IL-7 (R = 0.477, *p* = 0.046) (Table 3). 

Connective tissue IL-7 had a strong positive correlation with connective tissue IL-6 (R = 0.661, *p* = 0.002) and connective tissue IL-4 (R = 0.654, *p* = 0.002) (Table 2) and a moderate positive correlation with connective tissue IL-12 (R = 0.593, *p* = 0.007) and connective tissue Ki-67 (R = 0.483, *p* = 0.036) (Table 3).

### 3.5. Interleukin 8

A similar distribution was also seen with IL-8, where numerous-to-abundant (+++/++++) positive structures were in the epithelium and few (+) positive connective tissue structures were seen in the control samples (Figure 2 and Figure 3e), and the nasal polyp samples showed few (+) positive structures in the epithelium and moderate (++) numbers of positive structures in the subepithelial connective tissue (Figure 2 and Figure 4e). 

Epithelial IL-8 had a moderate positive correlation with epithelial IL-6 (R = 0.528, *p* = 0.020), epithelial IL-1 (R = 0.526, *p* = 0.021), epithelial IL-10 (R = 0.518, *p* = 0.023), and connective tissue IL-10 (R = 0.457, *p* = 0.049) (Table 2). 

Connective tissue IL-8 had a moderate positive correlation with connective tissue IL-10 (R = 0.530, *p* = 0.019) (Table 3). 

### 3.6. Interleukin 10

The number of IL-10 positive structures was noted with abundant (++++) cells in the epithelium and moderate (++) cells in the connective tissue of the control samples (Figure 2 and Figure 3f), and few (+) cells in the epithelium and moderate-to-numerous (++/+++) cells in the connective tissue of the nasal polyp samples (Figure 2 and Figure 4f).

Epithelial IL-10 had a strong positive correlation with epithelial Ki-67 (R = 0.797, *p* < 0.001), epithelial IL-1α (R = 0.699, *p* = 0.001), connective tissue Ki-67 (R = 0.640, *p* = 0.003), and epithelial IL-4 (R = 0.636, *p* = 0.003) (Table 2) and a moderate positive correlation with connective tissue IL-4 (R = 0.552, *p* = 0.014), epithelial IL-12 (R = 0.540, *p* = 0.017), epithelial IL-6 (R = 0.539, *p* = 0.017), epithelial IL-8 (R = 0.518, *p* = 0.023), and connective tissue IL-12 (R = 0.509, *p* = 0.026) (Table 3).

Connective tissue IL-10 had a moderate positive correlation with connective tissue IL-8 (R = 0.530, *p* = 0.019) and epithelial IL-8 (R = 0.457, *p* = 0.049) (Table 3).

### 3.7. Interleukin 12

Numerous (+++) IL-12 positive structures were noted in the epithelial tissue of the control samples, along with few (+) positive structures in the subepithelial connective tissue (Figure 2 and Figure 3g). The nasal polyp samples contained moderate (++) IL-12 positive structures in the epithelial cells and numerous (+++) positive structures in the connective tissue (Figure 2 and Figure 4g).

IL-12 in the epithelium had a very strong positive correlation with epithelial IL-6 (R = 0.826, *p* < 0.001) (Table 1), a strong positive correlation with epithelial IL-1α (R = 0.796, *p* < 0.001), epithelial IL-7 (R = 0.771, *p* < 0.001), epithelial Ki-67 (R = 0.730, *p* < 0.001), epithelial IL-4 (R = 0.706, *p* = 0.001), and connective tissue IL- 6 (R = 0.615, *p* = 0.005) (Table 2) and a moderate positive correlation with connective tissue IL-10 (R = 0.577, *p* = 0.010), connective tissue Ki-67 (R = 0.568, *p* = 0.011), connective tissue IL-4 (R = 0.545, *p* = 0.016), and epithelial IL-10 (R = 0.540, *p* = 0.017) (Table 3).

Connective tissue IL-12 had a strong positive correlation with epithelial IL-1 α (R = 0.740, *p* < 0.001) and connective tissue IL-1α (R = 0.696, *p* = 0.001) (Table 2) and a moderate positive correlation with connective tissue IL-7 (R = 0.593, *p* = 0.007), epithelial IL-6 (R = 0.559, *p* = 0.013), epithelial IL-7 (R = 0.547, *p* = 0.019), epithelial IL-10 (R = 0.509, *p* = 0.026), connective tissue Ki-67 (R = 0.496, *p* = 0.031), and connective tissue IL-6 (R = 0.494, *p* = 0.032) (Table 3). 

### 3.8. Proliferation marker Ki-67

Proliferation marker Ki-67 positive structures were observed in few-to-moderate (+/++) numbers in the epithelium and occasional (0/+) numbers in the subepithelial connective tissue in the case of the control samples (Figure 2 and Figure 3h). The nasal polyp samples displayed rare (0/+) Ki-67 positive structures in the epithelial cells and few positive structures in the connective tissue (Figure 2 and Figure 4h). 

Epithelial Ki-67 had a strong positive correlation with epithelial IL-10 (R = 0.797, *p* < 0.001), epithelial IL-12 (R = 0.730, *p* < 0.001), connective tissue Ki-67 (R = 0.728, *p* < 0.001), epithelial IL-4 (R = 0.653, *p* = 0.002), epithelial IL-1 α (R = 0.647, *p* = 0.003), and epithelial IL-6 (R = 0.647, *p* = 0.003) (Table 2) and a moderate correlation with epithelial IL-7 (R = 0.481, *p* = 0.043) (Table 3).

Connective tissue Ki-67 showed a strong positive correlation with epithelial IL-7 (R = 0.675, *p* = 0.002), epithelial IL-10 (R = 0.640, *p* = 0.003), epithelial IL-4 (R = 0.629, *p* = 0.004), and epithelial IL-1 (R = 0.625, *p* = 0.004) (Table 2) and a moderate correlation with epithelial IL-12 (R = 0.568, *p* = 0.011), epithelial IL-6 (R = 0.559, *p* = 0.013), connective tissue IL-4 (R = 0.532, *p* = 0.019), connective tissue IL-12 (R = 0.496, *p* = 0.031), and connective tissue IL-7 (R = 0.483, *p* = 0.036) (Table 3).

## 4. Discussion

We found a large number of correlations between factors in CRSwNP-affected mucosa samples. There were very strong, strong, and moderate correlations between factors in the tissue of recurrent nasal polyps. In samples of recurrent polyps, very strong correlations were detected between epithelial IL-6 and epithelial IL-7, IL-12, and IL-1α, thus helping us to conceptualize the role of IL-6. Cytokines displayed significant differences in the recurrent nasal polyp epithelium and connective tissue compared to the controls, with only non-significant differences in the tissue Ki-67 samples. Decreases in cytokines in the epithelial tissue and increases in the subepithelial connective tissue could be caused by a dysfunctional epithelial barrier that is already established as a characteristic feature of chronic rhinosinusitis with nasal polyps [3,15,16]. 

We observed a great number of correlations between IL-6 and other factors. Cytokines in the IL-6 group use a common receptor subunit glycoprotein 130 kDa (gp130) further on activating STAT3 and MAP signaling pathways. [17]. IL-6 and the IL-6 receptor (IL-6R) axis contribute to various autoimmune diseases, and their inhibition has shown considerable effects in such cases as rheumatoid arthritis [18]. IL-6 has already been linked to epithelial damage repair and cell proliferation in the pathogenesis of CRSwNP, therefore it is also evaluated here [19]. IL-6 is suggested to influence the Th17 (T-helper 17) and Treg (regulatory T cells) balance that has been associated with nasal polyps [20,21]. Indeed, IL-6 can promote the development of naive CD+ 4 T cells in Th17 cells and suppress the forkhead box P3 (Foxp3) expression, limiting Treg cell development, which allows for Th17 differentiation [22]. This suggests IL-6’s strong involvement in nasal polyp pathogenesis. IL-6 has been shown to influence wound repair in in vitro studies and may further lead to cell proliferation observed in nasal polyps [19]. This could be supported by our findings of strong and moderate correlations between both the epithelial and connective tissue of IL-6 and both the epithelial and connective tissue of Ki-67, as well as a very strong correlation between IL-6 and IL-1α. Therefore, IL-6 seems to be involved in epithelial repair as well as cell proliferation, since IL-1α is functioning as a key alarmin in the case of cell death, and Ki-67 characterizes proliferation. 

Our findings suggest a close relationship between IL-6 and IL-7 in the epithelium as well as in the subepithelial connective tissue of nasal polyps. IL-7 is a 25 kDa globular protein that is produced by bone marrow, stromal cells, thymus, and other epithelial cells. The IL-7 receptor, or IL-7R, consists of an α chain (CD127) and a γ chain that are shared with receptors of other cytokines. IL-7-mediated signaling is associated with JAK1, JAK3, and phosphoinositide 3-kinase (PI3K) that further activate STAT5 through phosphorylation [9]. Due to its role in T and B cell development, IL-7 is worth examining in the tissue of nasal polyps. IL-7 levels are thought to increase in cases of chronic inflammatory diseases such as inflammatory bowel or Chron’s disease, yet it has not been extensively evaluated in chronic rhinosinusitis [23]. IL-7R is expressed on naive as well as memory T cells, therefore, it can be presumed that active local adaptive immune responses are taking place in the tissue of nasal polyps that are somewhat facilitated by or due to the IL-6 expression. This interaction seems to take place in the epithelium as well as in the subepithelial connective tissue. These findings are based on very strong correlations between epithelial IL-6 and epithelial IL-7, and strong correlations between connective tissue IL-6 and both epithelial and connective tissue IL-7. 

Epithelial IL-12 had a very strong correlation with epithelial IL-6 and a strong correlation with connective tissue IL-6. The IL-12 and IL-6 families utilize shared receptors and cytokine subunits and influence the outcomes of inflammatory diseases [22]. IL-12 serves as an important link between innate and adaptive immunity and favors the differentiation of T helper 1 cells. IL-12 is produced by dendritic cells in response to microbial pathogens [12,24]. It helps in forming a link between adaptive and innate immunity, induces the production of interferon-γ, and favors the differentiation of T helper 1 cells. The IL-12 receptor is composed of two chains, IL-12Rβ1 and IL-12Rβ2, that activate the JAK (1 and 2)-STAT pathway. Specific IL-12 cellular effects are caused by STAT4 activation [12]. Due to its heterodimeric structure, IL-12 possesses unique connections and functional interactions, unlike other cytokines [24]. IL-12 has not been extensively looked at in CRSwNP, and due to its interesting properties, it is worth evaluating in the context of its microbial pathogen role in the formation of nasal polyps. The relationship between these two cytokines could be a basis for presumptions that the IL-6 activity and the epithelial repair along with cell proliferation are caused by microbial pathogens. This is supported by evidence that intracellular Staphylococcus aureus is associated with chronic rhinosinusitis [25,26,27].

Epithelial IL-6 had a strong correlation with epithelial IL-4 and a moderate correlation with connective tissue IL-4. However, connective tissue IL-6 had a strong correlation with connective tissue IL-4 and a moderate correlation with epithelial IL-4. IL-4 along with IL-13 is one of the main cytokines driving type 2 inflammation in as much as 80% of patients with nasal polyps [28]. They share a common receptor subunit and a signaling pathway via the Janus kinase (JAK)-STAT6 pathway. The IL-4 receptor system (IL-4R) comprises type 1 and type 2 receptors. IL-4 can be produced by secondary lymphoid tissue, consequently to antigen presentation, or in the peripheral tissue due to stimuli from alarmins, Th2 cytokines, and epithelial cell-derived cytokines [29]. IL-4 actively contributes to type 2 inflammation by facilitating the activation of B cells and the production of immunoglobulins, as well as stimulating Th2 differentiation from naive T cells and delaying their apoptosis after activation [30]. IL-4 along with IL-13 are responsible for increased epithelial permeability, thus allowing for more allergen exposure and causing CRSwNP signature symptoms, such as nasal discharge and oedema. Targeted therapy of monoclonal antibodies has been shown to control CRSwNP better than conventional intranasal corticosteroids [2,29]. IL-4 is also involved in stimulating CC chemokine TRAC release from fibroblasts of nasal mucosa and nasal polyps that, in turn, facilitate Th2 cell migration [31]. Due to the absence of IL-4R signaling, reduced IL-10 production occurs due to lack of IL-10+ Th2 cells [32]. This explains our findings of strong correlations between epithelial IL-10 and both connective tissue and epithelial IL-4. The relationship between IL-4 and IL-6 in our study is still unclear. Various authors have classified endotypes of chronic rhinosinusitis type 2 inflammation with predominant eosinophilia which formed specific clusters of endotypes and neutrophilic inflammation which formed different clusters [33,34]. Tomassen et al. (2016) found 10 clusters. Interestingly, some of the clusters were associated with milder clinical signs, less occurrences of polyps, and comorbid asthma and were characterized by IL-6 and IL-8 predominance, yet as the occurrences of nasal polyps and asthma increased, type 2 inflammation markers, such as IL-5 along with IL-6 and IL-8, were seen within clusters. The final cluster, which was the most saturated with cases of nasal polyps and asthma, had the highest concentration of IL-5 along with high concentrations of IL-6 and IL-8 and had the presence of s. Aureus superantigen [35]. Turner et al. (2018) also found a cluster with high concentrations of IL-4 and IL-5 as well as IL-6 and IL-8 [36]. Interestingly, though as much as other studies showed clusters with both IL-6 and IL-8 increases, we observed only a moderate correlation between epithelial IL-6 and IL-8. It could be speculated that the presumed CRS endotypes could be stages in inflammation with the same characteristics. 

Besides IL-6, epithelial IL-1α had strong correlations with various factors without a distinctive pattern, and it could be presumed that it functions as an alarmin and has no specific role in inflammation that advances the formation of nasal polyps. 

Ki-67 is a non-histone nuclear and nucleolar protein. It is localized in the peri-nucleolar region during the G1 phase and to the dense fibrillary component of the nucleolus during interphase. Therefore, it has been used as a tumor proliferation marker in various cancer types [37]. Ki-67 was significantly decreased in the epithelium of nasal polyps, although there were no differences in the connective tissue. Strong correlations between epithelial IL-4 and both epithelial and connective tissue Ki-67 suggest that IL-4 facilitates cell proliferation. This is supported by research on the increased Ki-67 release from nasal mucosal CD4+ T cells facilitated by IL-4. A strong relationship was observed in the cases of epithelial IL-10, IL-12, and IL-7, although the latter correlation was moderate. Some cytokines also demonstrated strong-to-moderate correlations with connective tissue Ki-67. Some studies have observed a higher Ki-67 concentration in nasal polyps [38,39]. Our findings indicate that the Ki-67 expression is not significantly changed in the case of CRSwNP when compared to the control samples, yet it characterizes IL-4, IL-7, IL-10, and IL-12 involvement in cell proliferation. 

Our findings are consistent with our previous work on primary CRSwNP. We observed similar characteristics of lower factor presence in the epithelium of polyps and a higher presence in the subepithelial connective tissue in comparison to the controls. This was true for the previous primary and now studied samples of the secondary CRSwNP mucosa. IL-6 demonstrated even more complex interactions with other factors, putting it as an important factor in recurrent CRSwNP pathogenesis. Ki-67 correlations with cytokines characterized previously established IL-4, IL-7, and IL-12 involvement in tissue proliferation in primary nasal polyps. Secondary CRSwNP showed the same Ki-67 correlations with an additional correlation with IL-10 in the case of recurrent nasal polyps [13]. 

Many previous authors have stated the importance of discovering molecular pathways or endotypes that could characterize chronic rhinosinusitis beyond phenotypes and aid us in better understanding and treating the disease. Many of the authors have classified their own CRS endotypes, yet all agree that further research is needed [33,34,35,36]. Novelty in our work lies in continuing to characterize inflammatory mechanisms in the case of CRSwNP in the Latvian population. We have found very strong indications of IL-6’s role in CRSwNP morphopatogenesis as well as novel interactions among Ki-67 and such cytokines as IL-4, IL-7, IL-10, and IL-12.

Our study has its fair share of limitations. In the future, we hope to associate our data with clinical parameters to give insights into their complex appearance, along with cytokines and Ki-67 in this disease. Further analysis with the ELISA method would be beneficial in evaluating precise cytokine concentrations because our data only shows immunohistochemical results. Further microbiological studies to evaluate intracellular S. Aureus would be beneficial.

## 5. Conclusions

IL-6 strongly correlates with other cytokines as well as with the proliferation marker Ki-67 which suggests significant stimulation of this regulatory cytokine and its possible involvement in the pathogenesis of recurrent nasal polyps. 

IL-4, IL-7, IL-10, and IL-12 correlate with Ki-67 which suggests the possible involvement of these cytokines in tissue cell proliferation in the case of recurrent nasal polyps. 

## Figures and Tables

**Table 1:** Very strong correlations among cytokines.

Factor 1	Factor 2	R	*p*-Value
Very strong correlation			
IL-6 in epithelium	IL-7 in epithelium	0.830 **	<0.001
IL-6 in epithelium	IL-12 in epithelium	0.826 **	<0.001
IL-1 in epithelium	IL-6 in epithelium	0.821 **	<0.001

*Abbreviations:* Ki-67—proliferation marker; IL-1—interleukin 1 alpha; IL-4—interleukin 4; IL-6—interleukin 6; IL-7—interleukin 7; IL-8—interleukin 8; IL-10—interleukin 10; IL-12—interleukin 12; **—correlation is significant at the 0.01 level.

**Table 2:** Strong correlations among cytokines and proliferation marker Ki-67.

Factor 1	Factor 2	R	*p*-Value
Strong correlation			
Ki-67 in epithelium	IL-10 in epithelium	0.797 **	<0.001
IL-1 in epithelium	IL-12 in epithelium	0.796 **	<0.001
IL-4 in epithelium	IL-6 in epithelium	0.781 **	<0.001
IL-7 in epithelium	IL-12 in epithelium	0.771 **	<0.001
IL-1 in epithelium	IL-12 in connective tissue	0.740 **	<0.001
Ki-67 in epithelium	IL-12 in epithelium	0.730 **	<0.001
Ki-67 in epithelium	Ki-67 in connective tissue	0.728 **	<0.001
IL-4 in epithelium	IL-12 in epithelium	0.706 **	0.001
IL-1 in epithelium	IL-10 in epithelium	0.699 **	0.001
IL-4 in epithelium	IL-7 in epithelium	0.698 **	0.001
IL-1 in connective tissue	IL-12 in connective tissue	0.696 **	0.001
IL-1 in epithelium	IL-7 in epithelium	0.690 **	0.002
IL-1 in epithelium	IL-4 in epithelium	0.688 **	0.001
Ki-67 in connective tissue	IL-7 in epithelium	0.675 **	0.002
IL-1 in epithelium	IL-4 in connective tissue	0.675 **	0.002
IL-6 in connective tissue	IL-7 in epithelium	0.663 **	0.003
IL-6 in connective tissue	IL-7 in connective tissue	0.661 **	0.002
IL-4 in connective tissue	IL-6 in connective tissue	0.658 **	0.002
IL-4 in connective tissue	IL-7 in connective tissue	0.654 **	0.002
Ki-67 in epithelium	IL-4 in epithelium	0.653 **	0.002
Ki-67 in epithelium	IL-1 in epithelium	0.647 **	0.003
Ki-67 in epithelium	IL-6 in epithelium	0.647 **	0.003
IL-4 in epithelium	IL-4 in connective tissue	0.647 **	0.003
Ki-67 in connective tissue	IL-10 in epithelium	0.640 **	0.003
IL-4 in epithelium	IL-10 in epithelium	0.636 **	0.003
Ki-67 in connective tissue	IL-4 in epithelium	0.629 **	0.004
Ki-67 in connective tissue	IL-1 in epithelium	0.625 **	0.004
IL-6 in connective tissue	IL-12 in epithelium	0.615 **	0.005

*Abbreviations:* Ki-67—proliferation marker; IL-1—interleukin 1 alpha; IL-4—interleukin 4; IL-6—interleukin 6; IL-7—interleukin 7; IL-8—interleukin 8; IL-10—interleukin 10; IL-12—interleukin 12; **—correlation is significant at the 0.01 level.

**Table 3:** Moderate correlations among cytokines and proliferation marker Ki-67.

Factor 1	Factor 2	R	*p*-Value
Moderate correlation			
IL-7 in connective tissue	IL-12 in connective tissue	0.593 **	0.007
IL-1 in epithelium	IL-1 in connective tissue	0.580 **	0.009
IL-12 in epithelium	IL-12 in connective tissue	0.577 **	0.010
Ki-67 in connective tissue	IL-12 in epithelium	0.568 *	0.011
Ki-67 in connective tissue	IL-6 in epithelium	0.559 *	0.013
IL-6 in epithelium	IL-12 in connective tissue	0.559 *	0.013
IL-4 in connective tissue	IL-10 in epithelium	0.552 *	0.014
IL-4 in epithelium	IL-6 in connective tissue	0.550 *	0.015
IL-7 in epithelium	IL-12 in connective tissue	0.547 *	0.019
IL-4 in connective tissue	IL-12 in epithelium	0.545 *	0.016
IL-10 in epithelium	IL-12 in epithelium	0.540 *	0.017
IL-6 in epithelium	IL-10 in epithelium	0.539 *	0.017
Ki-67 in connective tissue	IL-4 in connective tissue	0.532 *	0.019
IL-4 in connective tissue	IL-7 in epithelium	0.530 *	0.024
IL-8 in connective tissue	IL-10 in connective tissue	0.530 *	0.019
IL-6 in epithelium	IL-8 in epithelium	0.528 *	0.020
IL-1 in epithelium	IL-8 in epithelium	0.526 *	0.021
IL-8 in epithelium	IL-10 in epithelium	0.518 *	0.023
IL-10 in epithelium	IL-12 in connective tissue	0.509 *	0.026
Ki-67 in connective tissue	IL-12 in connective tissue	0.496 *	0.031
IL-6 in connective tissue	IL-12 in connective tissue	0.494 *	0.032
IL-6 in epithelium	IL-6 in connective tissue	0.485 *	0.035
Ki-67 in connective tissue	IL-7 in connective tissue	0.483 *	0.036
Ki-67 in epithelium	IL-7 in epithelium	0.481 *	0.043
IL-7 in epithelium	IL-7 in connective tissue	0.477 *	0.046
IL-4 in connective tissue	IL-6 in epithelium	0.468 *	0.043
IL-8 in epithelium	IL-10 in connective tissue	0.457 *	0.049

*Abbreviations:* Ki-67—proliferation marker; IL-1—interleukin 1 alpha; IL-4—interleukin 4; IL-6—interleukin 6; IL-7—interleukin 7; IL-8—interleukin 8; IL-10—interleukin 10; IL-12—interleukin 12; **—correlation is significant at the 0.01 level; *—correlation is significant at the 0.05 level.

## Data Availability

The data presented in this study are available upon request from the corresponding author.
